# The use of ASET (Anti Staph Epidermidis Titre) in the diagnosis of ventriculo-atrial shunt infection

**DOI:** 10.1186/1743-8454-7-S1-S44

**Published:** 2010-12-15

**Authors:** Jacqueline Reaper, Sally Ann Collins, John McMullan, Roger Bayston

**Affiliations:** 1 Dept of Neurosurgery, Royal Hallamshire Hospital, Sheffield S10 2JF, UK; 2 BRIG, Division of Orthopaedic and Accident Surgery, Queen’s Medical Centre, Nottingham NG7 2UH, UK

## Background

Infection is an important and not infrequent complication for patients with shunted hydrocephalus, with a reported incidence of 3-20%. Shunt infections can present in a non-specific manner and can be difficult to diagnose. Inflammatory markers may in rare cases be normal and CSF culture can also be negative, especially in Staph epidermidis infection. Ventriculo – atrial shunt infections are particularly prone to presentation with subtle clinical features and diagnosis may be delayed as a result. Since 1972, a simple serological test has been available for diagnosis of VA shunt infection, but is not widely used.

## Materials and methods

Casenotes were reviewed. The method of ASET testing was as published previously. Briefly, serial dilutions of patient’s serum and controls were reacted with S epidermidis antigen at 4degC and the antibody titre recorded.

## Results

A 38-year old male with hydrocephalus secondary to a Dandy Walker cyst had a ventriculoperitoneal shunt inserted as a child. Following numerous revisions and replacements, the shunt was revised to a ventriculo-atrial shunt in June 2008. There was no growth on CSF culture at this time. Following this he was well for seven months but re-presented with severe headaches and occasional night sweats. ICP monitoring showed no evidence of raised pressure. He was readmitted one month later complaining of night sweats and back pain, in addition to ongoing headaches. He was apyrexial, white cell count was normal and CRP was 30mg/L. CSF from lumbar puncture was sterile. Blood cultures grew Staphylococcus epidermidis, which our microbiologists thought to be a contaminant. However, shunt infection could not be excluded, blood was sent for Anti Staph Epidermidis Titre (ASET). Antibiotics were then started. ASET was >40,000 (normal range 160-640), confirming our suspicions of shunt infection. The shunt was removed, at which time, CSF and shunt tip grew S epidermidis. A new ventriculopleural shunt was inserted following a course of antibiotics. His headaches resolved and he was well following this.

## Conclusions

Our patient presented with non-specific symptoms. Initial CSF culture was normal and his symptoms were thought not to be due to infection. However, ASET confirmed S epidermidis shunt infection. This was then successfully treated and his symptoms completely resolved.

